# Supplementary material for: Identification of two pathways mediating protein targeting from ER to lipid droplets
Source: Nat Cell Biol. 2022 Sep 1;24(9):1364–77. doi: 10.1038/s41556-022-00974-0 (PMC9481466; doi:10.1038/s41556-022-00974-0)
Supplement: Supplementary file 1 — Reporting Summary [file 41556_2022_974_MOESM1_ESM.pdf]

nature portfolio

Corresponding author(s): Robert V. Farese, Jr. and Tobias C. Walther
Last updated by author(s): Jun 18, 2022

Reporting Summary

Nature Portfolio wishes to improve the reproducibility of the work that we publish. This form provides structure for consistency and transparency in reporting. For further information on Nature Portfolio policies, see our Editorial Policies and the Editorial Policy Checklist.

Statistics

For all statistical analyses, confirm that the following items are present in the figure legend, table legend, main text, or Methods section.

n/a	Confirmed	
☐	☒	The exact sample size (*n*) for each experimental group/condition, given as a discrete number and unit of measurement
☐	☒	A statement on whether measurements were taken from distinct samples or whether the same sample was measured repeatedly
☐	☒	The statistical test(s) used AND whether they are one- or two-sided<br>*Only common tests should be described solely by name; describe more complex techniques in the Methods section.*
☒	☐	A description of all covariates tested
☐	☒	A description of any assumptions or corrections, such as tests of normality and adjustment for multiple comparisons
☐	☒	A full description of the statistical parameters including central tendency (e.g. means) or other basic estimates (e.g. regression coefficient) AND variation (e.g. standard deviation) or associated estimates of uncertainty (e.g. confidence intervals)
☐	☒	For null hypothesis testing, the test statistic (e.g. *F*, *t*, *r*) with confidence intervals, effect sizes, degrees of freedom and *P* value noted<br>*Give P values as exact values whenever suitable.*
☒	☐	For Bayesian analysis, information on the choice of priors and Markov chain Monte Carlo settings
☒	☐	For hierarchical and complex designs, identification of the appropriate level for tests and full reporting of outcomes
☐	☒	Estimates of effect sizes (e.g. Cohen's *d*, Pearson's *r*), indicating how they were calculated

Our web collection on statistics for biologists contains articles on many of the points above.

Software and code

Policy information about availability of computer code

Data collection	NIS-Elements (4.51.01), Image Lab (6.0.1 build 34), IN Cell Analyzer 6000 Acquisition Software v1.0
Data analysis	Fiji (ImageJ 2.3.0/1.53f51), GraphPad Prism 7, MATLAB 9.2, Imaris (9.8.2), MaxQuant (1.5.2.8), Perseus (1.5.1.6)<br>Original code (used for quantification of screen images) has been deposited at Harvard Dataverse and is publicly available (DOI: https://doi.org/10.7910/DVN/NKJDWS).

For manuscripts utilizing custom algorithms or software that are central to the research but not yet described in published literature, software must be made available to editors and reviewers. We strongly encourage code deposition in a community repository (e.g. GitHub). See the Nature Portfolio guidelines for submitting code & software for further information.

Data

Policy information about availability of data

All manuscripts must include a data availability statement. This statement should provide the following information, where applicable:

- Accession codes, unique identifiers, or web links for publicly available datasets
- A description of any restrictions on data availability
- For clinical datasets or third party data, please ensure that the statement adheres to our policy

Original screen images and quantification results are available at the Lipid Droplet Knowledge Portal (http://lipiddroplet.org/; currently available to reviewers through http://3.92.40.39/fly_gene). The mass spectrometry proteomics data have been deposited to the ProteomeXchange Consortium via the PRIDE partner repository with the dataset identifier PXD027283. UniProt Drosophila melanogaster database.

Field-specific reporting

Please select the one below that is the best fit for your research. If you are not sure, read the appropriate sections before making your selection.

☒ Life sciences ☐ Behavioural & social sciences ☐ Ecological, evolutionary & environmental sciences

For a reference copy of the document with all sections, see nature.com/documents/nr-reporting-summary-flat.pdf

Life sciences study design

All studies must disclose on these points even when the disclosure is negative.

Sample size	No sample size calculation was performed. Genome-wide screen was repeated twice. Other experiments were repeated 2-3 independent times with 10-15 observations (cells) each.
Data exclusions	Outlier analysis was performed using ROUT method at Q = 1% on GraphPad Prism.
Replication	Number of independent experiments are indicated in the respective figure legends. No experiment was excluded in the analysis.
Randomization	The design of the RNAi assay plates for the genome-wide screen was randomized by the Drosophila RNAi Screening Center at Harvard Medical School. For all other cell experiments, randomization was not relevant/not performed, and the control and test conditions were performed side-by-side on the same day using the same reagents except for the treatment tested (such as RNAi or transfection).
Blinding	The RNAi assay plates for the genome-wide screen was prepared by the Drosophila RNAi Screening Center at Harvard Medical School, and the gene targets were not cross-referenced until all automatized analysis was completed. For all other cell experiments, blinding was not relevant/not performed.

Reporting for specific materials, systems and methods

We require information from authors about some types of materials, experimental systems and methods used in many studies. Here, indicate whether each material, system or method listed is relevant to your study. If you are not sure if a list item applies to your research, read the appropriate section before selecting a response.

Materials & experimental systems

n/a	Involved in the study	
☐	☒	Antibodies
☐	☒	Eukaryotic cell lines
☒	☐	Palaeontology and archaeology
☒	☐	Animals and other organisms
☒	☐	Human research participants
☒	☐	Clinical data
☒	☐	Dual use research of concern

Methods

n/a	Involved in the study	
☒	☐	ChIP-seq
☒	☐	Flow cytometry
☒	☐	MRI-based neuroimaging

Antibodies

Antibodies used	Rabbit anti-Drosophila GPAT4 (Dr. Tobias Walther, USA), Rat anti-Drosophila LDAH (Dr. Mathias Beller, Germany), Rabbit anti-Drosophila CCT1 (Dr. Tobias Walther, USA), Mouse anti-Drosophila CNX99A (Developmental Studies Hybridoma Bank, Cat# Cnx99A 6-2-1), Mouse anti-α-tubulin (Sigma Aldrich, Cat# T5168), Rabbit anti-Drosophila Sec16 (Dr. Catherine Rabouille, Netherlands), Guinea pig anti-Drosophila Tango1 (Dr. Sally Horne-Badovinac, USA), Mouse anti-rabbit IgG-HRP (Santa Cruz Biotechnology, Cat# sc-2357), Mouse anti-IgG kappa binding protein-HRP (Santa Cruz Biotechnology, Cat# sc-516102), Goat anti-rat IgG H&L-HRP (Abcam, Cat# ab97057), Alexa Fluor 647 goat anti-rabbit IgG (Thermo Scientific, Cat# A-21244), Alexa Fluor 488 goat anti-guinea pig IgG (Thermo Scientific, Cat# A-11073)
Validation	Rabbit anti-Drosophila GPAT4 (Wilfling et al., Dev Cell, 2013; validated with RNAi in Drosophila cells), Rat anti-Drosophila LDAH (Thiel et al., JCS, 2013; validated with RNAi in Drosophila cells), Rabbit anti-Drosophila CCT1 (Krahmer et al., Cell Metab, 2011; validated with RNAi in Drosophila cells), Mouse anti-Drosophila CNX99A (Riedel et al., Biology Open, 2016; validated with RNAi in Drosophila cells), Mouse anti-α-tubulin (Sigma Aldrich, Cat# T5168; Independent Antibody Verification – Demonstrating antibody specificity through the use of multiple antibodies against target in IHC or ICC), Rabbit anti-Drosophila Sec16 (Ivan et al., MBoC, 2008; validated with RNAi in Drosophila cells), Guinea pig anti-Drosophila Tango1 (Lerner et al., Dev Cell, 2013; validated with RNAi in Drosophila), Mouse anti-rabbit IgG-HRP (Santa Cruz Biotechnology, Cat# sc-2357; validated with western blot), Mouse anti-IgG kappa binding protein-HRP (Santa Cruz Biotechnology, Cat# sc-516102; validated with western blot), Goat anti-rat IgG H&L-HRP (Abcam, Cat# ab97057; validated with western blot), Alexa Fluor 647 goat anti-rabbit IgG (Thermo Scientific, Cat# A-21244; validated with immunofluorescence), Alexa Fluor 488 goat anti-guinea pig IgG (Thermo Scientific, Cat# A-11073; validated with immunofluorescence)

Eukaryotic cell lines

Policy information about cell lines

Cell line source(s)	Drosophila S2R+ cells used in the study were obtained from Dr. Norbert Perrimon (Harvard Medical School) and can be purchased from Drosophila Genomics Resource Center (stock number 150). Additional cell lines were created using CRISPR-Cas9 system as noted in the methods (endogenous EGFP knock-in cells to GPAT4 locus; seipin knockout cells)
Authentication	S2R+ cells were not authenticated. The generated cell lines were verified with western blot and mass spectrometer-based proteomics.
Mycoplasma contamination	All cell lines tested negative for mycoplasma.
Commonly misidentified lines (See ICLAC register)	No commonly misidentified cell lines were used.
