# Supplementary material for: Identification of two pathways mediating protein targeting from ER to lipid droplets
Source: Nat Cell Biol. 2022 Sep 1;24(9):1364–77. doi: 10.1038/s41556-022-00974-0 (PMC9481466; doi:10.1038/s41556-022-00974-0)

Fig. 4d original blots

Anti-GPAT4 (predicted MW: 43.6 kDa)

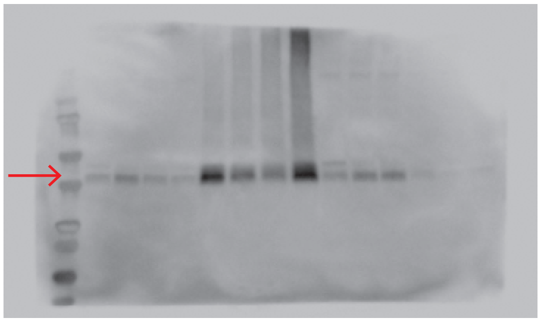

Ladder

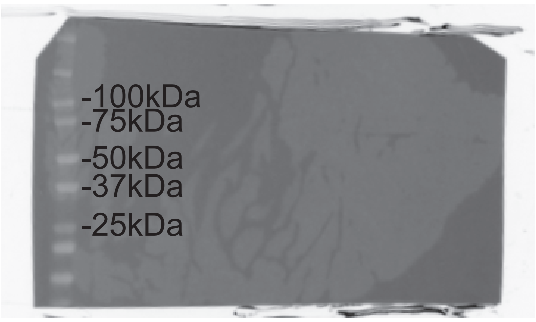

Anti-LDAH (predicted MW: 35.7 kDa)

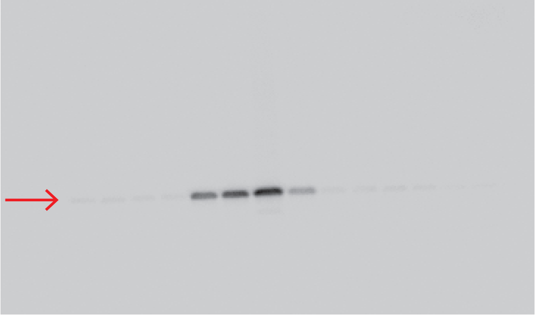

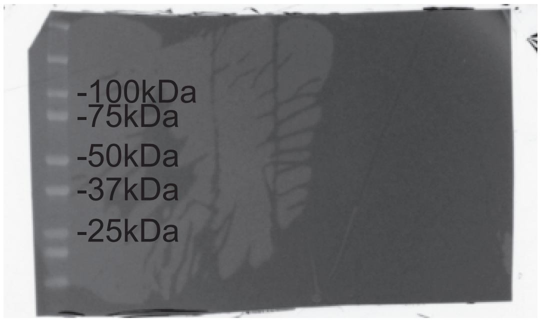

Anti-α-tubulin (predicted MW: 49.9 kDa)

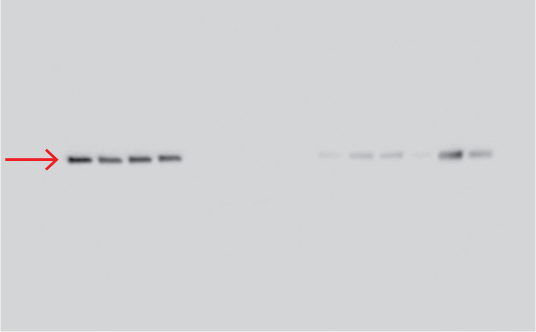

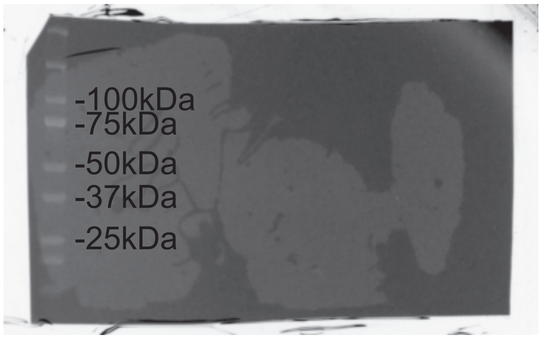

Anti-CNX99A (predicted MW: 75.9 kDa); note α-tubulin bands (~50kDa)

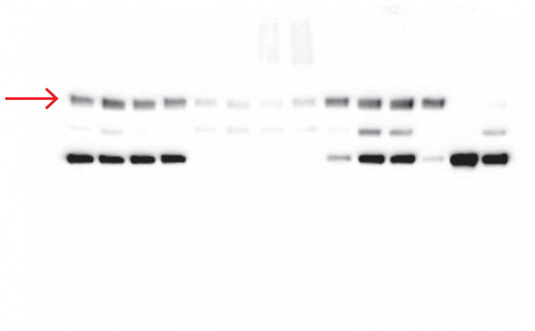

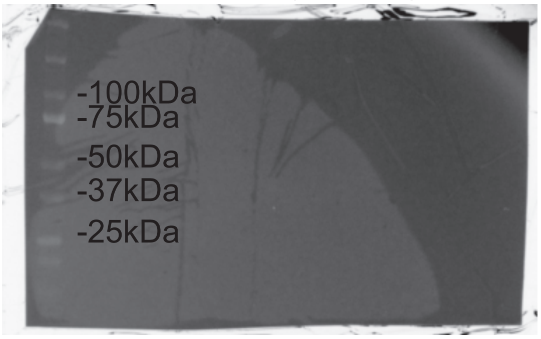

Anti-CCT1 (predicted MW: 58.2 kDa); note α-tubulin bands (~50kDa)

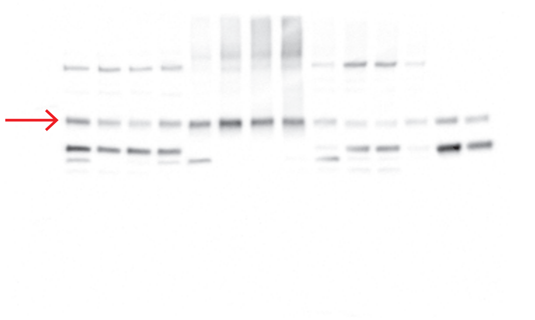

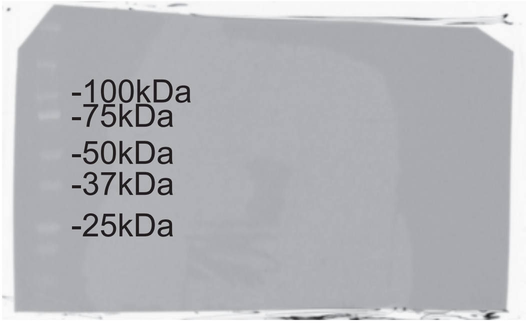

Supplement: Source Data Fig. 4 — Unprocessed western blots. [file 41556_2022_974_MOESM14_ESM.pdf]
