# Supplementary figures and images for: Identification of two pathways mediating protein targeting from ER to lipid droplets
Source: Nat Cell Biol. 2022 Sep 1;24(9):1364–77. doi: 10.1038/s41556-022-00974-0 (PMC9481466; doi:10.1038/s41556-022-00974-0)

Extended Data Fig. 5a original blots

Anti-GPAT4 (predicted MW: 43.6 kDa)

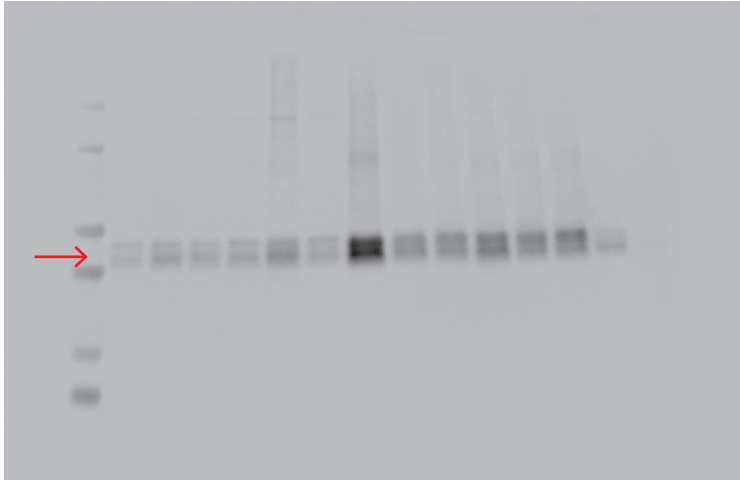

Ladder

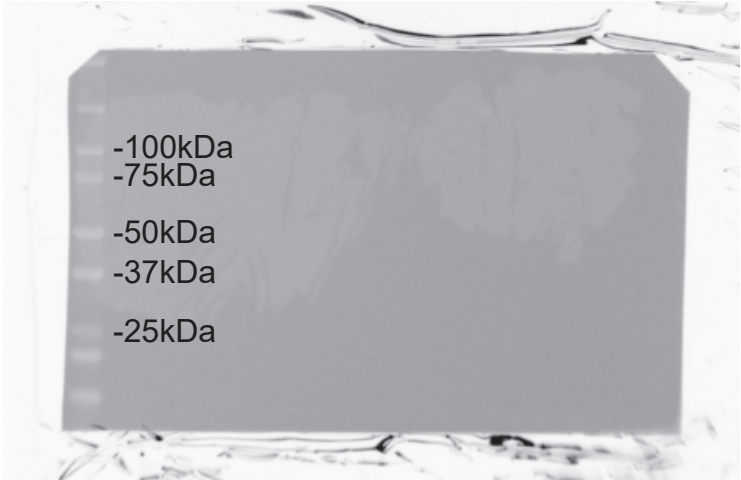

Anti-CNX99A (predicted MW: 75.9 kDa)

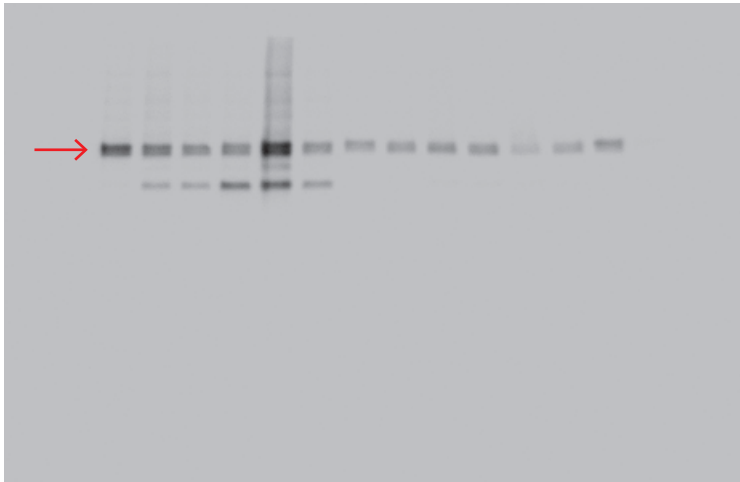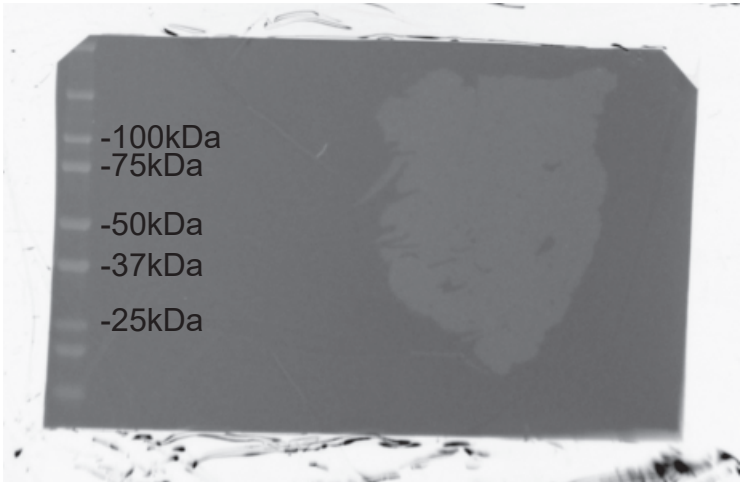

Supplement: Source Data Extended Data Fig. 5 — Unprocessed western blots. [file 41556_2022_974_MOESM23_ESM.pdf]
